# Clinical features distinguish cerebral amyloid angiopathy-associated convexity subarachnoid haemorrhage from suspected TIA

**DOI:** 10.1007/s00415-019-09558-9

**Published:** 2019-10-03

**Authors:** Joel Elliot Dane Stanton, Arvind Chandratheva, Duncan Wilson, Isabel Charlotte Hostettler, Saiful Islam, David John Werring

**Affiliations:** 1 Institute of Neurology Stroke Research Centre UCL, University College London, London, UK; 2 Stroke Research Centre, University College London, London, UK; 3 Department of Statistical Science, UCL Institute of Neurology, University College London, London, UK; 4 Department of Brain Repair and Rehabilitation, UCL Stroke Research Centre, UCL Queen Square Institute of Neurology, Russell Square House, 10-12 Russell Square, London, WC1B 5EH UK

**Keywords:** Cerebral amyloid angiopathy, Non-traumatic convexity subarachnoid haemorrhage, Convexial subarachnoid haemorrhage, Transient ischaemic attack, Mimic

## Abstract

**Objective:**

To identity clinical features that distinguish between cerebral amyloid angiopathy (CAA)-associated convexity subarachnoid haemorrhage (cSAH) and suspected TIA.

**Methods:**

We undertook a single-centre, retrospective case–control study. We identified cases [patients with cSAH presenting with transient focal neurological episodes (TFNE)] from radiological and clinical databases of patients assessed at the National Hospital for Neurology and Neurosurgery and UCLH Comprehensive Stroke Service. We identified age- and gender-matched controls at a 1:4 ratio from a database of consecutive suspected TIA clinic attendances at UCLH. We compared presenting symptoms and vascular risk factors between cases and controls.

**Results:**

We included 19 patients with cSAH-associated TFNE and 76 matched controls with suspected TIA. Migratory (spreading) symptoms (32% vs. 3%, OR 17.3; *p* = 0.001), sensory disturbance (47% vs. 14%, OR 5.3; *p* = 0.003,) and recurrent stereotyped events (47% vs. 19%, OR 3.7; *p* = 0.02,) occurred more frequently in patients with cSAH compared to controls. Hypercholesterolaemia was less common in patients with cSAH (16% vs 53%, OR 0.17; *p* = 0.008).

**Conclusion:**

Simple clinical features could help distinguish cSAH-associated TFNE from suspected TIA, with relevance for investigation and management, including the use of antithrombotic drugs.

## Introduction

The diagnosis of TIA is challenging, in part because of the wide range of differential diagnoses [1]. In older individuals, non-traumatic convexity subarachnoid haemorrhage (cSAH) can closely mimic TIA, because it typically presents with transient focal neurological episodes (TFNE)  associated with cererbral amyloid angiopathy (CAA) [also sometimes termed ‘amyloid spells’]. CAA-associated TFNE can cause both “positive” and “negative” neurological symptoms (e.g., focal weakness or numbness) [2]. Although previous studies suggest that spreading onset of symptoms or recurrent stereotyped attacks are characteristic for CAA-associated TFNE, we are not aware of any systematic studies comparing their symptoms to those of suspected TIA. Correctly differentiating between TFNE associated with cSAH (related to CAA) and TIA (or other non-haemorrhagic syndromes) is crucial as optimal treatments are likely to differ. In TIA, antiplatelet agents should be started within 24 h [3], with an increasing use of dual antiplatelet therapy in minor stroke or TIA [4], but in cSAH associated with CAA antithrombotic therapy should probably be avoided due to an increased risk of intracerebral haemorrhage [5]. Although CT can often identify acute cSAH [6], and MRI with blood-sensitive sequences readily detects cSAH and markers of CAA (including superficial siderosis and cerebral microbleeds) [2], simple clinical features could be helpful in guiding further investigations and treatment in patients with suspected CAA-associated TFNE during the acute phase of evaluation.

In this study, we aimed to identify clinical features that might help distinguish between CAA-associated TFNE (due to cSAH), and suspected TIA without evidence of any intracranial haemorrhage.

## Methods

We conducted a single-centre, retrospective case–control study. Cases with cSAH presenting with TFNE (defined according to a previous study) and attributed to CAA were identified through searching radiological and clinical databases of patients assessed at the UCLH Comprehensive Stroke Service and National Hospital of Neurology and Neurosurgery using the terms ‘convexity subarachnoid haemorrhage’ and ‘cortical subarachnoid haemorrhage’, between 2009 and 2015. cSAH presence was confirmed on CT and/or MRI imaging for all cSAH patients using brain imaging performed as part of standard care. cSAH was defined by a trained observer (DW, IH) as acute haemorrhage limited to the subarachnoid space over the convexities of the brain and not extending into the parenchyma, Sylvian fissures, ventricles, or basal cisterns. Control group patients underwent brain imaging as appropriate as part of standard care (CT and/or MRI as determined by the treating consultant). Demographic information (age, sex) was obtained from medical records for case–control matching. The control group consisted of four controls per case, matched by age and gender, selected from consecutive attendees with suspected TIA assessed at the UCLH TIA clinic during 2015–2016.

### Ethical approval

This study including only routine clinical data was approved by University College London Hospitals NHS Foundation Trust as a service evaluation.

### Data collection

Presenting symptoms and vascular risk factors were identified from electronic medical records. We systematically collected information on the following symptom characteristics: migratory (defined as any symptoms which spread to another body part from the time of onset, typically over a period of minutes); recurrent stereotyped events (defined as more than one substantially identical event within 4 weeks); sensory disturbance (including any changes in sensation, including positive or negative symptoms); weakness; speech disturbance; cheiro-oral symptoms (involving the hand and face in a single attack); paraesthesias (typically reported as “pins and needles” or “tingling”); headache; and visual disturbance (including either visual loss or positive visual symptoms). Some clinical syndromes would be included in more than one symptom category: for example, spreading paraesthesias affecting the mouth and hand would be included in migratory symptoms, sensory symptoms, paraesthesias, and cheiro-oral symptoms.

### Statistical analysis

Presenting symptoms and risk factors were compared between cases and controls. A univariate model of analysis was used to calculate *p* value, odds ratio and 95% confidence interval for each symptom and risk factor. We also conducted a post hoc multivariable analysis analysing whether spreading symptoms are associated with cSAH independently from sensory symptoms, recurrent stereotyped events and visual disturbances, respectively.

Statistical analysis was performed using SPSS [IBM Corporation, version 24.0.0] and STATA 15 (StataCorp. 2017. *Stata Statistical Software: Release 15*. College Station, TX: StataCorp LLC).

### Data availability

Anonymized data supporting the findings of this study will be made available to appropriately qualified investigators on request.

## Results

**Fig. 1:**
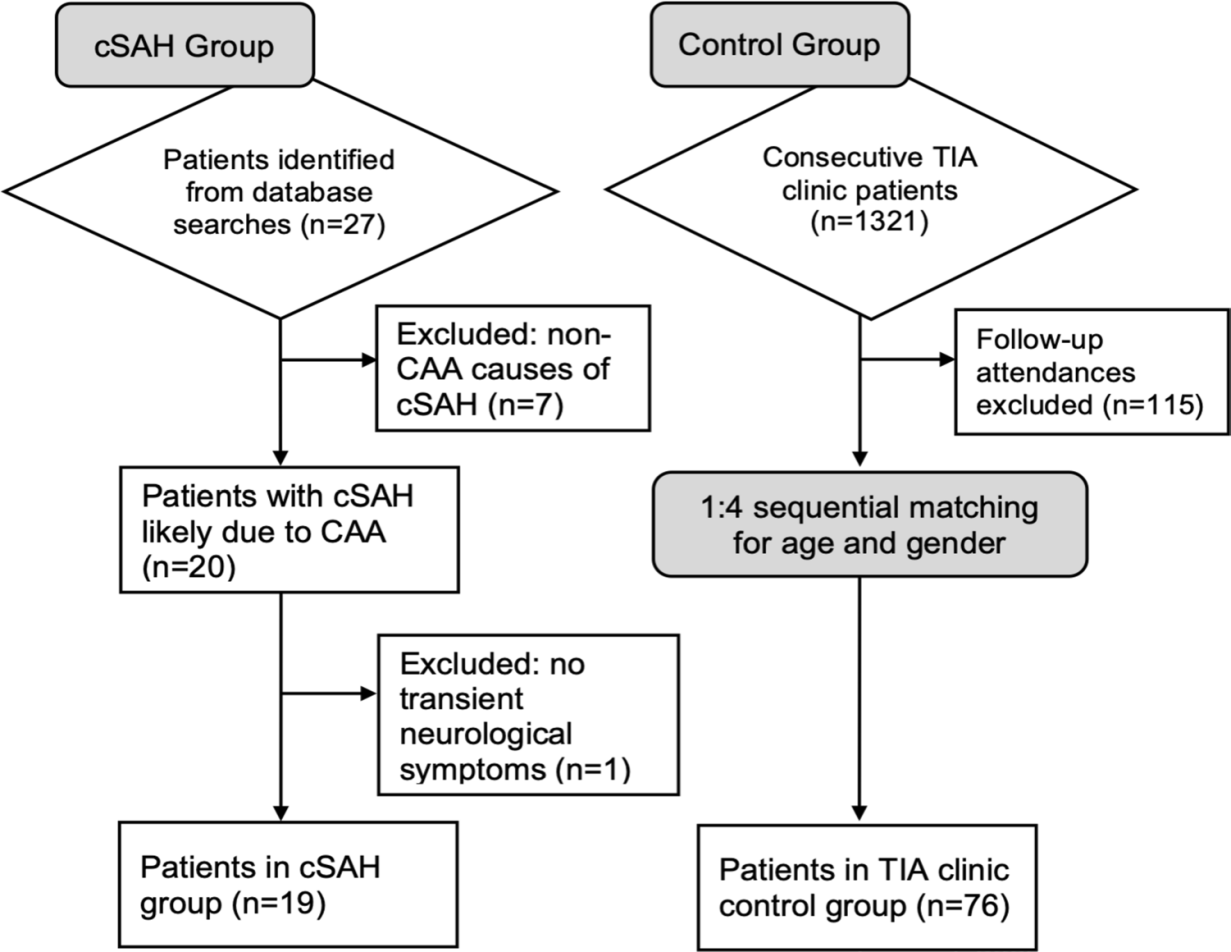
Flow diagram of patient selection

We identified 27 patients with cSAH due to suspected CAA. We excluded patients diagnosed with underlying causes of cSAH other than suspected CAA (*n* = 7), and those who did not present with TFNE (*n* = 1) (Fig. 1). Our final cohort included 19 patients with CAA-associated cSAH presenting with TFNE, and 76 matched controls (Table 1). In the control group with suspected TIA (*n* = 76), the final diagnoses were: TIA (*n* = 30, 40%), minor stroke (*n* = 10, 13%), migraine with aura (*n* = 8, 11%), ocular pathology (*n* = 7, 9%), vertigo (*n* = 3, 4%), seizure (*n* = 2, 3%), syncope (*n* = 2, 3%), radiculopathy (*n* = 2, 3%), arteriovenous malformation (*n* = 1, 1%), unspecified headache (*n* = 1, 1%), trigeminal neuralgia (*n* = 1, 1%), transient global amnesia (*n* = 1, 1%), functional (*n* = 1, 1%), and uncertain (*n* = 7, 9%). Subgroup data for controls with a final diagnosis of TIA are shown in Tables 1 and 2.

**Table 1:** Patient characteristics for cases (cSAH) and controls

	cSAH (*n* = 19)	Controls (*n* = 76)	Confirmed TIA subgroup (*n* = 30)	*p* value*	Univariable OR** (95% CI)
Age: mean (SD)	68.9 (9.03)	68.9 (8.9)	72.6 (7.76)	1.0	
Male sex: *n* (%)	11 (55)	44 (55)	19 (63)	1.0	
Atrial fibrillation	1 (5)	4 (5)	2 (7)	1.0	1.0 (0.1–9.5)
Excessive alcohol	0 (0)	2 (3)	0 (0)	0.9	0.8 (0.04–16.6)
Hypertension	10 (53)	54 (59)	20 (67)	0.1	0.5 (0.2–1.3)
Previous stroke/TIA	1 (5)	14 (18)	7 (23)	0.2	0.3 (0.03–2.0)
Smoker	0 (0)	5 (7)	4 (13)	0.5	0.3 (0.01–6.3)
Ex-smoker	5 (26)	20 (26)	5 (17)	1.0	1.0 (0.3–3.1)
Diabetes mellitus	0 (0)	8 (11)	2 (7)	0.3	0.2 (0.01–3.7)
Hypercholesterolaemia	3 (16)	40 (53)	18 (60)	0.008	0.17 (0.05–0.6)
Antithrombotic therapy, *n* (%)	5 (26)	24 (32)	11 (37)	0.7	0.8 (0.3–2.4)

*Difference in proportions for cSAH vs controls

**For cSAH vs controls

**Table 2:** Presenting symptoms in cases (cSAH) and controls

	cSAH (*n* = 19)	Controls (*n* = 76)	Confirmed TIA subgroup (*n* = 30)	*p* value*	Univariable OR** (95% CI)
Migratory (spreading) symptoms	6 (32)	2 (3)	0 (0)	0.001	17.3 (3.1–93.9)
Sensory disturbance	9 (47)	11 (14)	2 (7)	0.003	5.3 (1.8–16.0)
Recurrent stereotyped events	9 (47)	15 (19)	4 (13)	0.02	3.7 (1.3–10.6)
Paraesthesia	3 (16)	4 (5)	1 (3)	0.1	3.3 (0.7–16.6)
Cheiro-oral symptoms	2 (11)	0 (0)	0 (0)	0.4	2.1 (0.4–12.5)
Weakness	7 (37)	22 (29)	13	0.5	1.4 (0.5–4.1)
Speech disturbance	5 (26)	19 (25)	10 (33)	0.9	1.3 (0.3–3.4)
Visual disturbance	0 (0)	19 (25)	5 (17)	0.08	0.08 (0.004–1.3)

*Difference in proportions for cSAH vs controls

**For cSAH vs controls

**Fig. 2:**
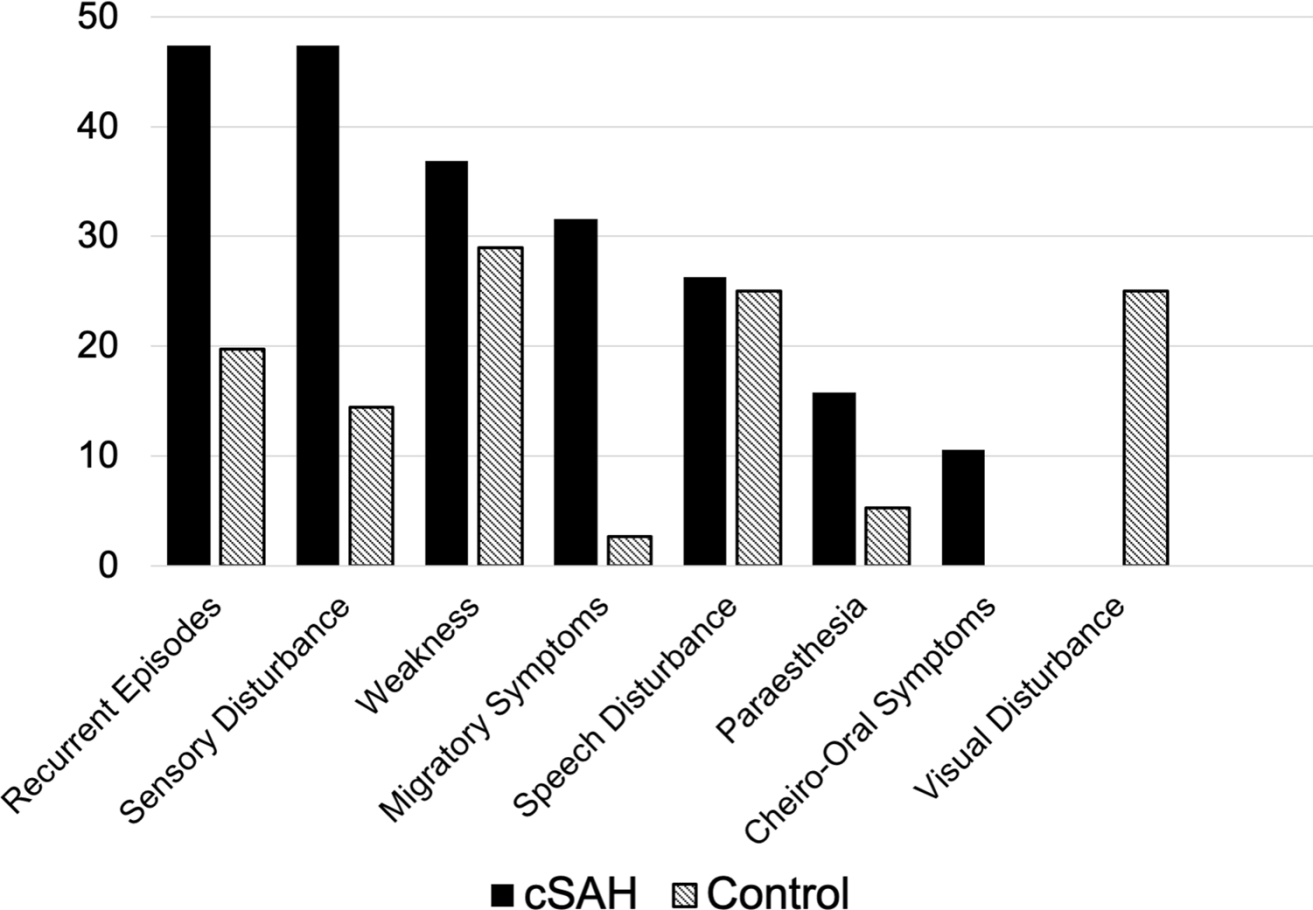
Bar chart showing the frequency of clinical features in cSAH cases and matched controls

Compared to controls, patients with cSAH-associated TFNE were more likely to present with migratory symptoms (32% vs 3%, OR 17.3; *p* = 0.001), sensory disturbance (47% vs. 14%, OR 5.3; *p* = 0.003), and recurrent stereotyped events (47% vs 19%, OR 3.7; *p* = 0.02,); those with cSAH had a lower prevalence of hypercholesterolaemia (16% vs 53%, *p* = 0.008, OR 0.17) (Fig. 2; Table 2).

In post hoc multivariable analyses, migratory symptoms and sensory disturbance were independently associated with cSAH, without evidence of interaction (*p* value for interaction = 0.7). It was not possible to assess the interaction between migratory symptoms and recurrent stereotyped events due to collinearity.

## Discussion

Our main finding is that distinct clinical features (including migratory symptoms and recurrent stereotyped events) are more likely in patients with cSAH due to suspected CAA than in a matched control group of patients with suspected TIA. Among vascular risk factors, hypercholesterolaemia was less common in those with CAA-associated cSAH compared to controls (16% vs 53% *p* = 0.008, OR 0.17).

Our findings are consistent with previous descriptions of TFNE associated with CAA [2], which include spreading onset and recurrent stereotyped attacks. Cortical depolarisation and cortical spreading depression [7] are likely mechanisms, but vasospasm and ischaemia have also been suggested. By contrast, migratory symptoms are not a common feature of TIA, with sensory or motor symptoms usually having sudden onset in a fixed, rather than spreading, territory [1].

Although both TIAs and CAA-associated cSAH can present with recurrent stereotyped events, this pattern seems much more common in cSAH; we found that almost half (47%) of our cohort with cSAH experienced two or more stereotyped episodes. Recurrent waves of cortical depolarisation or cortical spreading depression [7] or vasospasm secondary to a focal area of cSAH and potentially irritative blood products trapped within a cerebral sulcus might explain this observation. Although TIAs can be recurrent, stereotyped attacks are less common, probably because the commonest underlying mechanisms—namely, emboli from large arteries or cardiac sources—are not likely to repeatedly occlude the same artery to cause identical recurrent patterns of ischaemic injury and clinical features over a short time period.

Patients with CAA-associated cSAH were more likely to experience sensory disturbances compared to those with suspected TIA. This might be due to symptomatic cSAH often affecting the central sulcus, causing functional disturbance in the primary sensory cortex of the postcentral gyrus. We found that visual disturbance was not a feature of cSAH-associated TFNE in our cohort, while this was present in 25% of our TIA clinic controls (although this difference was not statistically significant, perhaps due to our limited sample size). Nevertheless, visual symptoms might also have diagnostic value for determining the likelihood of cSAH in patients presenting with transient neurological symptoms. Although sensory disturbance and recurrent stereotyped events were common in the cSAH group (each being seen in 47% of participants), their absence cannot be used clinically to reliably exclude cSAH.

Our finding of a lower prevalence of hypercholesterolaemia in those with cSAH is consistent with the established inverse relationship between cholesterol and both non-traumatic intracerebral ICH and aneurysmal SAH.

Our study has some limitations. We collected data retrospectively so that clinical features were extracted from routine clinical records rather than standardised prospective case report forms. The time period of ascertainment of cases and controls overlapped, but patients with cSAH-associated TFNE were seen over a longer time period and from different referral sources, which could have contributed to between-group differences. Because we did not do a prospective study, we cannot give an accurate prevalence for CAA-associated cSAH in a TIA clinic as not all TIA clinic patients in the control group underwent imaging if it was not clinically indicated. However, one recent paper suggested that this might be around 1% [8]. Our sample size remains small, with wide confidence intervals around the odds ratios for each presenting symptom. This study specifically investigated CAA-related acute cSAH and did not investigate symptoms associated with the non-acute finding of cortical superficial siderosis, which can also be associated with TFNE in patients with CAA. Thus, our findings cannot be generalised to patients with cortical superficial siderosis but no acute cSAH; we are not aware of data comparing the clinical features of patients with cSAH to those with only established cortical superficial siderosis.

Nevertheless, our findings strongly suggest that in patients presenting with transient neurological symptoms, simple clinical features can help to identify patients with cSAH-associated TFNE attributed to CAA, with value for planning early investigations (e.g., undertaking blood-sensitive MRI to seek markers of CAA) and treatment (e.g., avoiding acute administration of antithrombotic agents).
